# From Dust Devil to Sustainable Swirling Wind Energy

**DOI:** 10.1038/srep08322

**Published:** 2015-02-09

**Authors:** Mingxu Zhang, Xilian Luo, Tianyu Li, Liyuan Zhang, Xiangzhao Meng, Kiwamu Kase, Satoshi Wada, Chuck Wah Yu, Zhaolin Gu

**Affiliations:** 1 School of Human Settlements and Civil Engineering, Xi'an Jiaotong University, Xi'an, China; 2 Photonics Control Technology Team, RIKEN Center for Advanced Photonics, The Institute of Physical and Chemical Research, Wako-shi, Saitama, Japan; 3 International Society of the Built Environment (ISBE), Milton Keynes, UK

## Abstract

Dust devils are common but meteorologically unique phenomena on Earth and on Mars. The phenomenon produces a vertical vortex motion in the atmosphere boundary layer and often occurs in hot desert regions, especially in the afternoons from late spring to early summer. Dust devils usually contain abundant wind energy, for example, a maximum swirling wind velocity of up to 25 m/s, with a 15 m/s maximum vertical velocity and 5 m/s maximum near-surface horizontal velocity can be formed. The occurrences of dust devils cannot be used for energy generation because these are generally random and short-lived. Here, a concept of sustained dust-devil-like whirlwind is proposed for the energy generation. A prototype of a circular shed with pre-rotation vanes has been devised to generate the whirlwind flow by heating the air inflow into the circular shed. The pre-rotation vanes can provide the air inflow with angular momentum. The results of numerical simulations and experiment illustrate a promising potential of the circular shed for generating swirling wind energy via the collection of low-temperature solar energy.

A dust devil is a small whirlwind unique weather phenomenon of a vertically oriented rotating column of air on Earth and on Mars^1^,^2^, usually of short-lived duration, with a low pressure and warm core. The height of the dust column ranges from about several tens of metres to thousand metres^2^,^3^. Dust devils found in nature contain an enormous amount of wind energy and can easily raise dust, sand, and debris to high altitudes^3^,^4^,^5^,^6^,^7^.

A consensus has been reached on how dust devils are formed. The ambient vertical vorticity (angular momentum) combined with a vertical motion due to heat buoyancy (solar radiation) is the main mechanism for developing the phenomenon. When a net circulation of wind flow exists around the perimeter of an area, a buoyant convection within that area can occur that would strengthen the circulation and increase the local vorticity by stretching the wind flow^8^,^9^,^10^,^11^,^12^, as illustrated in Fig. 1a. Since both heat buoyancy and ambient vorticity tend to be unsustained in nature, the occurrences of dust devils are consequently random and short-lived.

There have been wide spread use of solar heat for power generation^13^,^14^,^15^,among them, the utilization of a buoyancy-induced flow is an important model. Power generation using spontaneous buoyancy-induced vortices^15^ is a new method. Here, we propose the use of a circular solar-energy-collecting shed with pre-rotation vanes to produce a steady dust-devil-like wind field to generate sustainable whirlwind energy. A schematic representation of the shed is shown in Fig. 1b. A swirling air mass can flow out of the central outlet, and form a sustainable and long-lived whirlwind (swirling buoyant jet). Consequently, low-temperature solar energy is transferred into swirling wind energy.

This study focuses on the inflows in the solar-energy-collecting shed and the swirling buoyant jet at the central outlet, to show the potential for swirling wind energy use. An internal mechanical model of the shed with eight vanes (Supplementary Figure 1a) was constructed for the experiment to realize the concept of dust-devil-like whirlwind depicted in Fig. 1b. The flow patterns and potential energy of the whirlwind produced by the system was investigated. The sensitivity analysis of the inlet vorticity was conducted, and the effects of temperature differences and geometrical parameters on the swirling buoyant jet were evaluated by computational fluid dynamics (CFD) numerical simulation and experimental tests.

## Results

### Velocity distribution

The inlet vorticity is a key factor in stimulating a whirlwind. Different inlet vorticities can be obtained with different air inflow incident angles through the vanes, then the effect of inlet vorticity on the buoyant jet air flow field was subsequently investigated by numerical modelling. The resultant velocity of the swirling buoyant jet (*U_mag_*) and its tangential velocity component (*U_τ_*) were selected to display the total kinetic energy and the swirling feature of the swirling buoyant jet, respectively. *U_mag_* is defined by Equation (1) as,
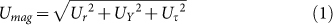
where *U_r_* is the radial velocity, *U_Y_* is the vertical velocity, and *U_τ_* is the tangential velocity.

**Figure 2:**
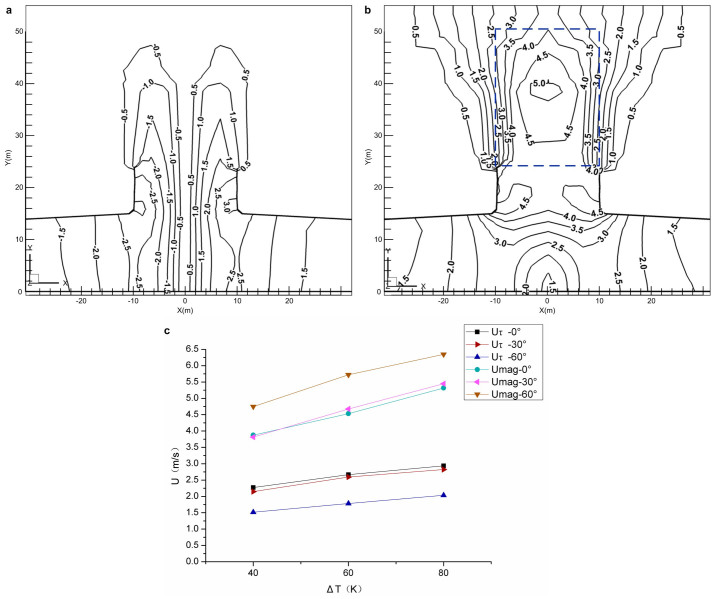
Local swirling buoyant jet in the shed with central induced duct and the velocity trend with the temperature difference and incident angle. (a), Contours of the tangential velocity component (swirling velocity) *U_τ_* are characteristic of the low-velocity core region, which are similar to that of a dust devil found in nature^20^. (b), Contours of the resultant velocity *U_mag_*, indicate the swirling wind velocity, which is up to 3 m/s in the buoyant jet (plotted by dashed lines), reaching the cut-in wind velocity for wind energy use. (c), Increase in the velocity due to the heating temperature difference with different incident angles (*R* = 200 m).

The local swirling buoyant jet for one of the cases, whose conditions are as follows (The other simulated cases are listed in Supplementary Table 1): the solar-energy-collecting shed radius, *R* = 200 m, the air inflow incident angle, *α*, at the inlet of the shed is 0° and the temperature difference, *ΔT*, between the heating source and ambient air is 80 K. The velocity distributions are shown in Fig. 2a and Fig. 2b. As can be seen, there is a wind region of over 3 m/s (plotted by dashed lines) occupied by the swirling buoyant jet. The cut-in velocity of a wind field for wind energy use is 3 m/s^16^. Therefore, a helical wind turbine entrained in the swirling buoyant jet can potentially be developed for whirlwind energy use.

### Sensitivity analysis

To demonstrate the effects of *ΔT* and *α* on the swirling buoyant jet, a large number of simulations were carried out in this research by varying one of the parameters while keeping the other physical modelling parameters constant. Detailed results are given in Supplementary Table 2. All the resultant velocity values when using *R* = 200 m for simulations exceeded 3 m/s, thus illustrating the possibility of whirlwind energy use. Therefore, a larger temperature difference would help to raise the velocity of the swirling buoyant jet, and to increase the wind energy output potential. The incident angle of the air inflow at the inlet of the solar-energy-collecting shed should be carefully chosen because of its opposite effect on the swirling velocity while positive effect on the resultant velocity (Fig. 2c).

### Experimental analysis

**Figure 3:**
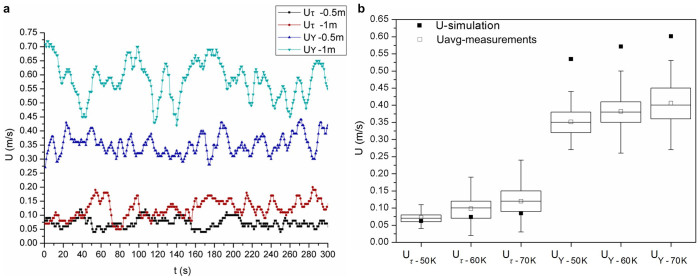
Modelling heating shed experiment. (a), Wind velocities for two different electric heating powers. Two different electric heating pads with *R* = 0.5 m and *R* = 1 m were investigated in the experiments with *ΔT* = 50 K. (b), Box-and-whisker diagram of the measured swirling velocity, *U_τ_*, and vertical velocity, *U_Y_* were respectively obtained by experiments in comparison with the corresponding simulation values. Each plot shows the statistical results of 300 measurement values. The top and bottom lines(whiskers) represent the highest and lowest values, and the three horizontal lines in the box represent the upper and lower quartiles and the median. The result shows the validity of the numerical simulation. Due to the ideal wall friction effect of the induced duct in the small-scale modelling of the heating shed by simulation, the simulated vertical velocity, *U_Y_* was greater than the average value measured in the experiment.

A heating shed model with *R* = 2 m (Supplementary Figure 1a), was set up to test the dust-devil like swirling buoyant jet. The experiments with *ΔT* = 50 K, 60 K, and 70 K were conducted. The swirling velocity and vertical velocity at two points were measured as marked in Supplementary Figure 1b. Measurements obtained from the experiment with *ΔT* = 50 K were shown in Fig. 3a as an illustration. These results were plotted as a box-and-whisker diagram and then compared with the simulation values, as illustrated in Fig. 3b. The swirling velocity and vertical velocity tended to increase with the heating temperature difference and this could aid the efficacy of the swirling wind energy generation. A video of the generated swirling wind was recorded and the wind was compared with a typical dust devil found in nature (Supplementary Video 1).

### Similarity-theory estimation

By considering the similarity theory of fluid dynamics, the appropriate size of solar-energy-collecting shed can be obtained for the available velocity of swirling wind field. From the theory, the same Froude number (*Fr*) would mean a similar wind flow can be produced by two different sizes of the shed with their corresponding swirling buoyant jets. For two corresponding swirling buoyant jets, the averaged resultant velocity of the swirling buoyant jet above the central outlet and the radius of the shed can be defined as their characteristic velocity and the characteristic length, respectively. The characteristic velocity and the characteristic length are two key parameters to evaluate the Froude number (Methods: Similarity-theory estimation method). It follows that the ratio of their characteristic velocities, *δ_u_*, is related to the ratio of their characteristic lengths, *δ_L_*, as represented by Equation (2) as follows,
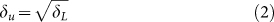


Therefore, the characteristic average resultant velocity of the swirling buoyant jet at the central outlet can be increased parabolically with the radius of the shed, as represented by Equation (3).
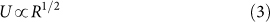


**Figure 4:**
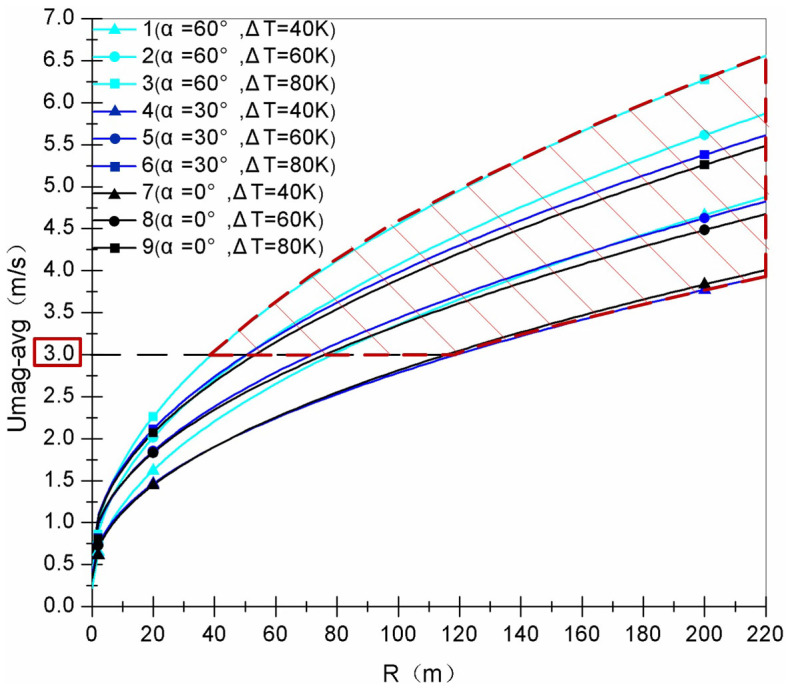
Regime of solar-energy-collecting shed size for the least cut-in velocity wind energy use. As a function of shed radii, the results were obtained by simulation with different radii of the solar-energy-collecting shed with a heating temperature difference of 40 K ≤ *ΔT* ≤ 80 K and an incident angle of 0° ≤ α ≤ 60°.

Following the cases listed in Supplementary Table 1, the variations of the characteristic averaged resultant velocities of the swirling buoyant jets with the shed radii are shown in Fig. 4. When the average velocity of the swirling buoyant jet at the central outlet is 3 m/s, which is the cut-in velocity for whirlwind energy use, the confines of the solar-energy-collecting shed size under different heating temperature differences (40 K, 60 K, and 80 K) and different incident angles (60°, 30°, and 0°) are illustrated in Fig. 4. The simulation provides information for the development of the swirling wind energy generation by this solar-energy-collecting shed.

## Discussion

Generally, a helical wind turbine with the blade curve normal to the flow stream of the wind field would be configured, using the total kinetic energy of the swirling wind, i.e., the resultant velocity *U_mag_*. The helical wind turbine is mounted in the upstream above the central shed, to utilize the swirling wind energy system driven by solar heat.

The thermodynamic fundamentals of the above swirling wind system is the temperature difference *ΔT* between the average temperature of the air inflow and the heating temperature of the solar-energy-collecting shed. High average temperature of the air inflow during daytime would require high heating temperature of the shed to keep a certain temperature difference, whereas the lower average temperature of the air inflow at nighttime would require lower heating temperature of the shed to maintain the same temperature difference. Generally, the collected solar energy is sensitive heat. According to the principle of energy cascade use, during daytime, part of the collected solar energy of high temperature would be directly utilized to heat the air inflow while part of the collected energy of low temperature would be stored by phase-change materials. The stored low-temperature solar energy can then be utilized to heat the low-temperature air inflow at nighttime. i.e., there might always be enough temperature difference to drive the air inflow both day and night during a desert summer.

In addition, if the top surface of the shed is covered by a solar panel system, a hybrid solar photovoltaic power-heat driven swirling wind energy system could potentially be developed. How would such a hybrid system work? Only a small part of the energy absorbed by the solar panel would be converted to electricity, and much of the absorbed solar energy would need to be removed, usually resulting in high temperature, more than 100°C, at the back of the solar panel^17^. Thus, the back of the solar panel could be used as the heating surface to heat the air inflow. This hybrid system would increase the comprehensive energy efficiency of solar energy. In reality, owing to poor ventilation, high temperature at the back of the solar panel would have a negative effect on the output voltage, energy conversion efficiency^18^ and even service life of the solar panel. The converting ratio of solar energy to the swirling wind total kinetic energy should be improved by further optimizing the structure of the system, this could contribute a significant role in future energy industry.

Similar to the wind field of dust devil in nature, there is a relatively low velocity at the center of the rising swirling flow, so installation of the shaft of the helical wind turbine at the center has little effect on swirling buoyant jet. Moreover, designing the new patterns of blades for swirling wind energy output is particularly significant to improve the operation of the system. To realize the artificial swirling wind energy system, the profile of solar-energy-collecting shed and its temperature distribution, the inlet vorticity and the profile of the induced duct should be further optimized based on the fundamental study of the fluid mechanics of the swirling buoyant jet.

## Methods

### Physical modeling

In this part, we discuss the parameters of the physical model (Supplementary Figure 2, for example) and the initial boundary conditions of the cases outlined in the paper.

The heating temperature differences and vane incident angle are key parameters for generating the swirling buoyant jet. Nine cases under different temperature (40 K, 60 K, and 80 K) and different angle (0°, 30°, and 60°) were simulated, respectively. The radius of the solar-energy-collecting shed used for the simulation were: 2 m, 20 m, and 200 m, see Supplementary Table 1.

### Governing equations

In our simulations, the *k-ε* two-equation model was adopted. For computational fluid dynamics (CFD) applications, the governing equations were solved in terms of the prescribed boundary and initial conditions. All the variants (velocities, temperature, turbulent kinetic energy, and dissipation) to be solved by the governing equations are denoted by *φ*. The general transport equation for *φ* is shown in Equation (4),
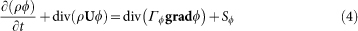


Where, *ρ* is the density of the fluid, **U** = (***u, v, w***) is the velocity vector, *Γ_*φ*_* is the generalized diffusion coefficient, and *S_φ_* is the source term. With properly prescribed *Γ_*φ*_*, *S_φ_* and *φ*, the equation can express the continuity, momentum, energy and other scalar equations^19^.

### Algorithm, boundary and initial conditions

For the computational domain, the rectangular grid subdivision (grid number) is about 327,430 in total with particularly refinement about the shed area. The PIMPLE (PISO + SIMPLE) algorithm was used to decompose pressure-velocity couple. In terms of the numerical scheme, the time discretization was performed through the first-order bounded implicit method, and the gradient and diffusion terms used were based on the second-order unbounded Gauss interpolation method.

The boundary and initial conditions are given in Supplementary Table 1. The temperature of the ambient environment was 273 K, and the pressure at the boundary was a standard atmospheric pressure. The open-source CFD code OpenFOAM (Field Operation and Manipulation) was implemented in the simulations.

### Detailed simulation analysis

All the simulation results are shown in Supplementary Table 2. The values in the table are the maximum velocities of the buoyant jet field, including the maximum tangential (swirling) velocity *U_τ_* and the maximum resultant velocity *U_mag_* which were used to study the relationship between velocity and *α or ΔT.* According to the results of the cases (*R* = 200 m and *α* = 0°), the swirling buoyant jet velocity changes with the temperature difference. The simulations with temperature differences of 40 K, 60 K, and 80 K show that the maximum swirling velocity and maximum resultant velocity increased simultaneously with the temperature difference. The maximum swirling velocity showed a 17% incremental increase with a temperature difference increasing from 40 K to 60 K. However, a 10% incremental increase was achieved with a temperature difference increasing from 60 K to 80 K, while the incremental increase in maximum resultant velocity remained at 17%. On the other hand, at the same temperature difference of 80 K, cases of the swirling buoyant jet with the incident angle 0°, 30°, and 60° were also simulated. Larger incident angle would mean less vorticity of the air inflow at the inlet of the shed. Therefore with smaller incident angle, an increase in the maximum swirling velocity was achieved but with a reduction in the maximum resultant velocity. When the incident angle was reduced from 60° to 30°, an incremental increase in the maximum swirling velocity was 39%. However, when the incident angle was reduced from 30° to 0°, an incremental increase of 4% was achieved, with a 14% to 2% reduction in the maximum resultant velocity.

Supplementary Figure 3 shows (a) the contours of the swirling velocity of the buoyant jet and (b) the contours of a dust devil in nature^20^ and also (c) low pressure regions at the center of the jet. These features are similar to the dust devils in nature.

### Experimental investigation

According to the physical model (*R* = 2 m), the mock-up of the shed was constructed to validate the result of numerical simulation (Supplementary Figure 1a).

The distance between the end of the each vane and the center of the shed is 1 m. For the convenience of recording videos, the middle of the shed is a circular perspex panel (*R* = 0.6 m), the radius of the central outlet is 0.1 m. Above the outlet is the cylindrical wall, the height is 0.5 m and the radius is 0.3 m, which was used to keep the flow stable and separate the inner flow from the ambient environment. Silicone rubber plate heating system was used to provide the heat source (in Supplementary Figure 1c).

For the control and measurement, in the plate heating system, the ED330L digital display temperature control device (precision: ±1°C, setting temperature range: 30–100°C) was used to maintain the heating temperature. The heating temperature was measured by the TM-902C thermocouple (range: −50–1300°C with a resolution ratio: 0.1°C). The wind velocity was measured by TSI9515 (temperature range: −18–93°C, velocity accuracy: ±5% or ±0.025 m/s whichever is greater). In Supplementary Figure 1b, two points, M and N were selected for measuring the vertical velocity *U_Y_* and tangential velocity *U_τ_*, respectively.

The following experimental procedure was conducted:

- After measuring the environmental temperature, the heating pad A (*R* = 0.5 m) was turned on, setting a temperature difference of 50 K, 60 K, and 70 K, respectively, and measuring the corresponding vertical velocity of point M and the tangential velocity of point N. Each test took 300 s and obtained 300 values.
- The heating pads A, B, and C (*R* = 1 m) were turned on, and repeated step (i).
- The heating pads A, B, and C (*R* = 1 m) were turned on, and set the heating temperature to 80°C. A video (Supplementary Video 1.) was recorded of the air flow in the shed from the vane's end to the central outlet (the whirlwind) with white smoke tracer.
- Obtained the statistical average velocities of different experiments, and then compared the experimental data with numerical simulations at points M and N, respectively.

On the experiment day the ambient air temperature was 13°C, the surface temperature of the heating pad was 18°C before heated.

When the heating temperature difference was 50 K, the velocities achieved under the heating pad A (the heating area, *R* = 0.5 m) and the heating pads A + B + C (the heating area, *R* = 1 m) are illustrated in the Fig. 3a, respectively.

Obviously, the wind velocity would be increased with an increase in the heating area (heating capacity). Under the heating pad A (the heating area, *R* = 0.5 m), the statistically averaged tangential and vertical velocities were obtained with heating temperature differences of 50 K, 60 K and 70 K; these are illustrated in Fig. 3b. The simulation results with the same conditions are shown in Fig. 3b.

Supplementary Video 1 shows the whirlwind flow at the central outlet in the experiment. In the video, a typical dust devil found in nature is shown.

### Similarity-theory estimation method

The flow in the shed and the swirling buoyant jet are essentially a heat driven buoyant flow. The decisive forces for buoyant flow have three elements: buoyant force, inertial force and viscous force. Particularly, for the turbulent buoyant flow, the viscous force could be negligible. So the *Fr* number and the ratio of inertial force to buoyant force were taken as the similarity criterion to study the scale effect of the solar-energy-collecting shed on the velocity field of the swirling buoyant jet. The *Fr* number is defined by Equation (5):
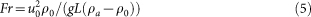


Where *u*_0_ is the average resultant velocity of the swirling buoyant jet above the central outlet; *ρ*_0_ is the airflow density; *g* is the gravitational acceleration; *L* is the radius of the solar-energy-collecting shed; and *ρ_a_* is the ambient air density.

From the similarity theory of fluid dynamics, the same *Fr* number means a similar flow in the solar-energy-collecting shed of two different sizes and their corresponding swirling buoyant jets.

## Author Contributions

Z.G. and X.L. conceived the steady dust devil-like whirlwind producing system and supervised the project. M.Z., X.M. and C.W.Y. fabricated the experimental device, performed numerical simulation, and prepared the manuscript. T.L. and L.Z. contributed to the design of the experimental system. K.K. and S.W. contributed to the numerical simulation and the manuscript revision.

## Supplementary Material

Supplementary Information Video of the generated swirling wind and the comparison with a typical dust devil found in nature

Supplementary Information Supplementary Information for

## Figures and Tables

**Figure 1:**
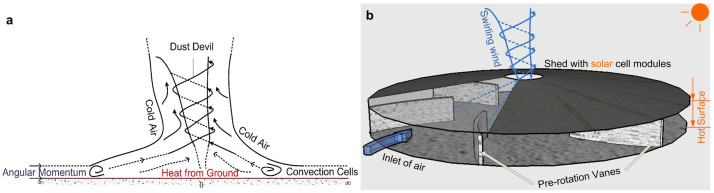
Dust devil occurring in nature, and configuration of a steady dust devil-like whirlwind producing system. (a), Evolution of a dust devil in nature. The near-surface air layer, heated by the hot ground (heat driven by solar radiation), becomes warm which may gradually lead to instability since this warm air layer is covered with a layer of cold air. The layer of air cannot turn around completely, thus, a convective plume forms at the center of the rising convection. As the convective plume rises to higher altitudes, the low pressure at its base deepens. This near-surface low-pressure center forces a spiral inflow of warm boundary layer air into the incipient dust devil. The swirling energy increases at the center, and then a dust devil emerges. (b), Design of the solar-energy-collecting shed for steady swirling airflows. The circular shed has pre-rotation vanes around the perimeter. As the shed is heated, the surrounding air flowing through the channels between the pre-rotation vanes becomes a swirling buoyant jet at the central outlet.
