# Supplementary material for: From Dust Devil to Sustainable Swirling Wind Energy
Source: Sci Rep. 2015 Feb 9;5:8322. doi: 10.1038/srep08322 (PMC4321178; doi:10.1038/srep08322)
Supplement: Supplementary Information — for [file srep08322-s2.pdf]

Supplementary Information for

From Dust Devil to Sustainable Swirling Wind Energy

Mingxu Zhang[1], Xilian Luo[1*], Tianyu Li[1], Liyuan Zhang[1], Xiangzhao Meng[1], Kiwamu Kase[2], Satoshi Wada[2], Chuck Wah Yu[3], Zhaolin Gu[1*]

Affiliations:

[1]School of Human Settlements and Civil Engineering, Xi'an Jiaotong University, Xi'an, China

[2]Photonics Control Technology Team, RIKEN Center for Advanced Photonics, The Institute of Physical and Chemical Research, Wako-shi, Saitama, Japan

[3]International Society of the Built Environment (ISBE), Milton Keynes, UK

[*]Corresponding author. Email: xlluo@mail.xjtu.edu.cn; guzhaoln@mail.xjtu.edu.cn

Supplementary Figure 1-Figure 3

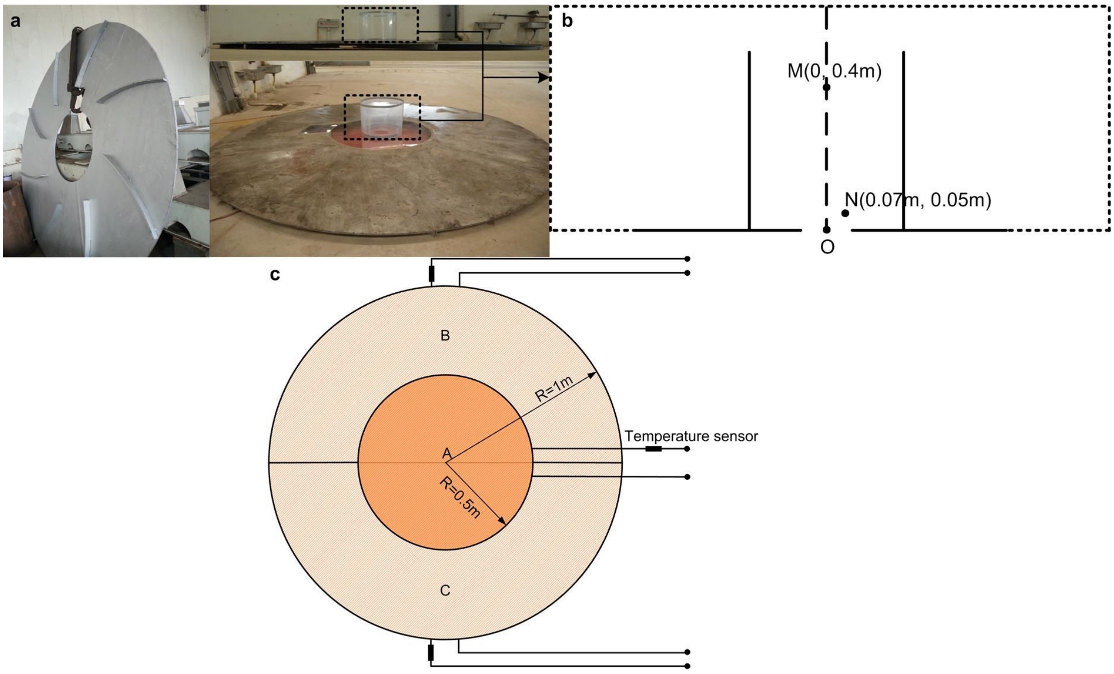

Supplementary Figure 1 | Experimental facilities. a, Mock-up of the shed with eight vanes and 60° incident angle. **b**, Measuring point allocations. **c**, Allocation of two different areas of electric heating pads with R = 0.5 m and R = 1 m, respectively.

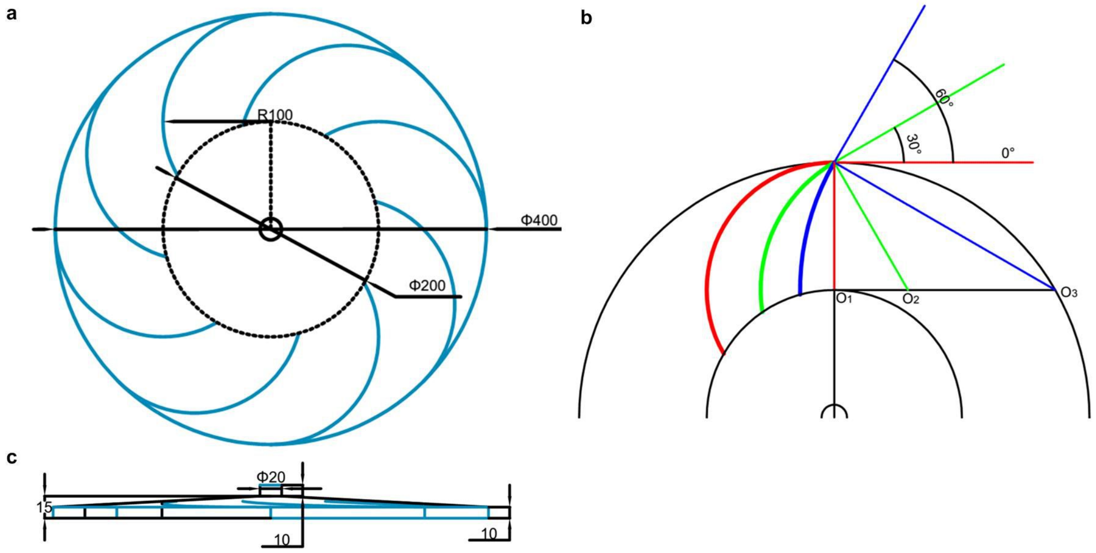

Supplementary Figure 2 | Modelling heating shed with eight vanes. a, Vertical view of the shed. (unit: m) **b**, Vane allocation with different air inflow incident angles

α. **c**, Front view of the shed. The top surface of the shed has a slope, and an induced duct is installed at the central outlet to ensure the stability of the swirling buoyant jet. (unit: m)

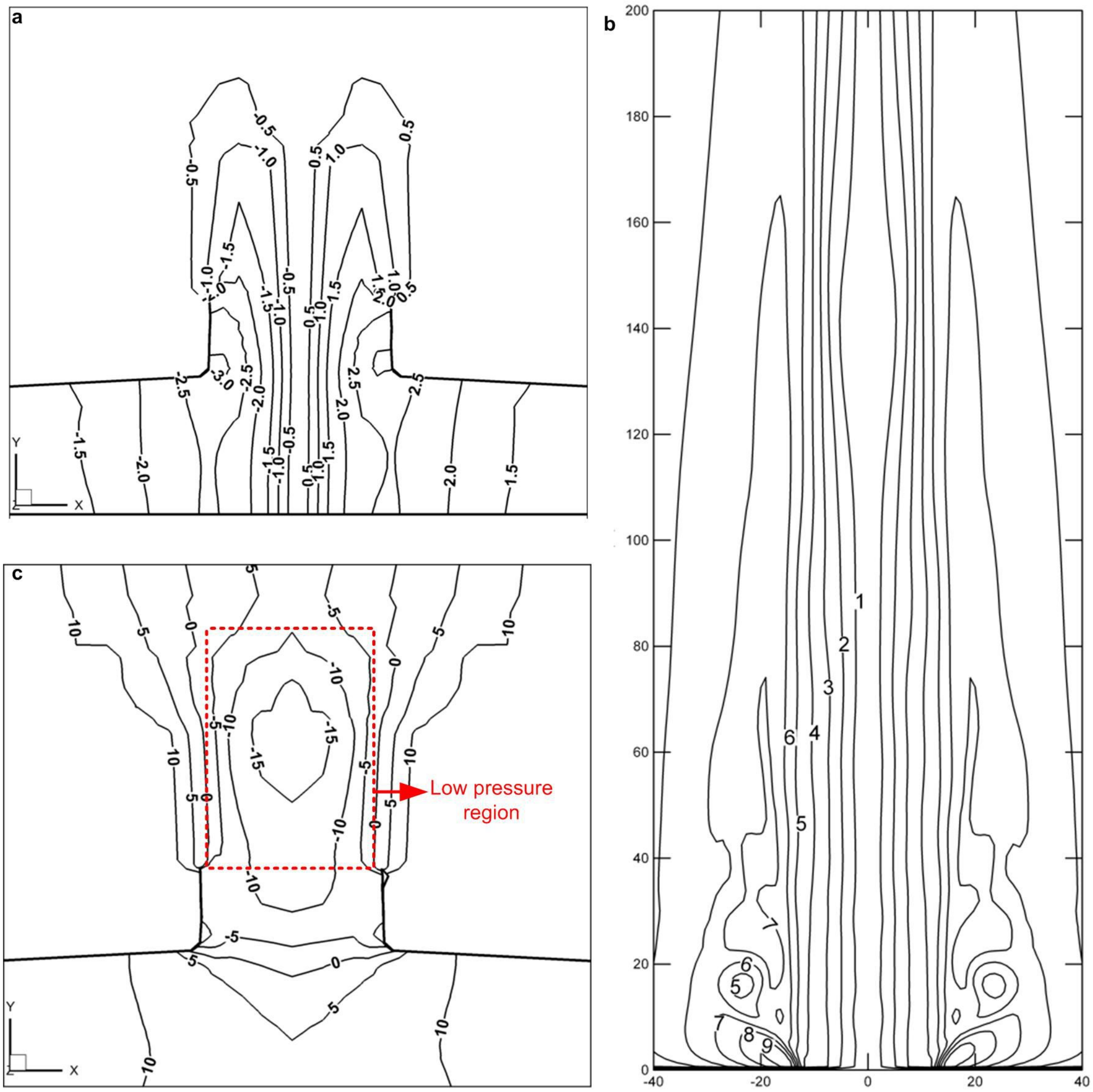

Supplementary Figure 3 | Comparison of the contours between a swirling buoyant jet and a dust devil found in nature. a, Contours of swirling velocity (unit: m/s) for shed radius R = 200 m, air inflow incident angle α = 0°, and temperature difference ΔT = 80 K. **b**, Swirling velocity contours of a typical dust devil found in

nature. **c**, Contours of pressure distribution showing the low pressure region at the center of the jet (unit: Pa).

Supplementary Table 1-Table 2

Supplementary Table 1 | Structure parameters and temperature differences in the simulations

Case 1	R=2m/α=60°/ΔT=40K	R=20m/α=60°/ΔT=40K	R=200m/α=60°/ΔT=40K
Case 2	R=2m/α=60°/ΔT=60K	R=20m/α=60°/ΔT=60K	R=200m/α=60°/ΔT=60K
Case 3	R=2m/α=60°/ΔT=80K	R=20m/α=60°/ΔT=80K	R=200m/α=60°/ΔT=80K
Case 4	R=2m/α=30°/ΔT=40K	R=20m/α=30°/ΔT=40K	R=200m/α=30°/ΔT=40K
Case 5	R=2m/α=30°/ΔT=60K	R=20m/α=30°/ΔT=60K	R=200m/α=30°/ΔT=60K
Case 6	R=2m/α=30°/ΔT=80K	R=20m/α=30°/ΔT=80K	R=200m/α=30°/ΔT=80K
Case 7	R=2m/α=0°/ΔT=40K	R=20m/α=0°/ΔT=40K	R=200m/α=0°/ΔT=40K
Case 8	R=2m/α=0°/ΔT=60K	R=20m/α=0°/ΔT=60K	R=200m/α=0°/ΔT=60K
Case 9	R=2m/α=0°/ΔT=80K	R=20m/α=0°/ΔT=80K	R=200m/α=0°/ΔT=80K

Supplementary Table 2 | Swirling velocity and characteristic resultant velocity of each case

	R = 2m		R = 20m		R = 200m	
	U_τ(m/s)	U_{mag}(m/s)	U_τ(m/s)	U_{mag}(m/s)	U_τ(m/s)	U_{mag}(m/s)
Case 1	0.147	0.702	0.593	1.648	1.516	4.748
Case 2	0.181	0.823	0.681	2.053	1.784	5.723
Case 3	0.205	0.926	0.775	2.304	2.036	6.345
Case 4	0.204	0.663	0.790	1.482	2.148	3.814
Case 5	0.260	0.868	0.937	1.879	2.593	4.675
Case 6	0.297	0.928	1.027	2.138	2.824	5.446
Case 7	0.206	0.677	0.834	1.464	2.274	3.874
Case 8	0.293	0.780	0.961	1.854	2.665	4.532
Case 9	0.308	0.873	1.057	2.094	2.937	5.316
